# Influence of In Vitro IL-2 or IL-15 Alone or in Combination with Hsp-70-Derived 14-mer Peptide (TKD) on the Expression of NK Cell Activatory and Inhibitory Receptors

**DOI:** 10.1155/2013/405295

**Published:** 2013-02-17

**Authors:** Ilona Hromadnikova, Petra Pirkova, Lucie Sedlackova

**Affiliations:** Department of Molecular Biology and Cell Pathology, Third Faculty of Medicine, Charles University in Prague, Ruska 87, 100 00 Prague, Czech Republic

## Abstract

NK cells represent a potential tool for adoptive immunotherapy against tumors. Membrane-bound Hsp70 acts as a tumor-specific marker enhancing NK cell activity. Using flow cytometry the effect of in vitro stimulation with IL-2 or IL-15 alone or in combination with Hsp70-derived 14-mer peptide (TKD) on cell surface expression of NK activatory receptors (CD16, NKG2D, NKG2C, NKp46, NKp44, NKp30, KIR2DL4, DNAM-1, and LAMP1) and NK inhibitory receptors (NKG2A, KIR2DL2/L3, LIR1/ILT-2, and NKR-P1A) in healthy individuals was studied. Results were expressed as the percentage of receptor expressing cells and the amount of receptor expressed by CD3^−^CD56^+^ cellular population. CD94, NKG2D, NKp44, NKp30, KIR2DL4, DNAM-1, LAMP1, NKG2A, and NKR-P1A were upregulated after the stimulation with IL-2 or IL-15 alone or in combination with TKD. KIR2DL2/L3 was upregulated only by IL-15 and IL-15/TKD. Concurrently, an increase in a number of NK cells positive for CD94, NKp44, NKp30, KIR2DL4, and LAMP1 was observed. IL-15 and IL-15/TKD caused also cell number rise positive for KIR2DL2/L3 and NKR-P1A. Cell number positive for NKG2C and NKG2A was increased only by IL-2 and IL-2/TKD. The diverse effect of IL-2 or IL-15 w or w/o TKD on cell surface expression was observed in CD16, NKp46, and LIR1/ILT-2.

## 1. Introduction

Hsp70 has been shown to play an active role in oncogenic transformation and be abundantly expressed in most cancer cells [1]. Plasma membrane Hsp70 was demonstrated to act as a tumor-specific recognition structure for preactivated NK cells expressing high amounts of CD94. Furthermore, the amount of membrane-bound Hsp70 correlated with sensitivity to lysis mediated by NK cells [2, 3]. Gastpar et al. [4] explore the effect of the Hsp70-derived peptide TKD in the stimulation of resting NK cells against Hsp70 membrane-positive tumors. Incubation of peripheral blood lymphocytes with TKD peptide plus a low dose of IL-2 initiates the cytolytic and migratory capacity of NK cells toward Hsp70-membrane-positive tumor cells in vitro and in a xenograft tumor mouse model [5]. In a recently published clinical phase I trial, the tolerability, feasibility, and safety of IL-2/TKD-activated NK cells were tested in patients with colorectal and lung carcinomas [6, 7]. 

The goal of the current study was to determine the effect of in vitro stimulation using IL-2 and/or IL-15 alone or in combination with stress-inducible Hsp70-derived 14-mer peptide (TKD) on cell surface expression of NK cell activatory and inhibitory receptors in peripheral blood mononuclear cells of healthy individuals. The cell surface expression profile of the following activatory receptors was studied on NK cell population using flow cytometry: a low affinity receptor for aggregated IgG (CD16), members of NKG2 natural killer cell receptor family (NKG2D/CD314 and NKG2C associating with CD94 to form a heterodimer), members of the natural cytotoxicity receptor (NCR) family (NCR1/NKp46, NCR2/NKp44, and NCR3/NKp30), a killer cell immunoglobulin-like receptor (KIR2DL4/CD158d), DNAX accessory molecule-1 (DNAM-1/CD226), and lysosome-associated membrane protein-1 (LAMP1/CD107a).

Additionally, the cell surface expression of the following NK cell inhibitory receptors was determined: NKG2A creating a complex with CD94 molecule, a killer cell immunoglobulin-like receptor (KIR2DL2/L3/CD158b, NKAT2), a member of the leukocyte immunoglobulin-like receptor (LIR) family such as the immunoglobulin-like transcript 2 (LIR1/ILT-2/CD85j), and a killer cell lectin-like receptor subfamily B, member 1 (KLRB1) also known as NKR-P1A/CD161.

## 2. Materials and Methods

Peripheral blood mononuclear cells (PBMC) were isolated by density gradient centrifugation using Ficoll-Paque (Amersham Biosciences, Little Chalfont, UK) from remaining fresh buffy coats after plasma separation from blood donors sampled in Department of Blood Transfusion, University Hospital Kralovske Vinohrady, Prague. Local ethics committee approval was obtained prior testing.

PBMC at concentration of 5 × 10^6^ were cultured in 5 mL RPMI 1640 medium (Cambrex Bio Sciences Verviers, Verviers, Belgium) supplemented with heat-inactivated 5% (v/v) fetal calf serum (FCS, Sigma Biosciences, St. Louis, MO, USA), 6 mM L-glutamine (Gibco Invitrogen Corporation, Carlsbad, CA, USA), and antibiotics (100 U/mL penicillin and 100 *μ*g/mL streptomycin, Sigma Biosciences, St. Louis, MO, USA) at 37°C in a humidified atmosphere of 5% CO_2_ in the presence or absence of recombinant human cytokines IL-2 (100 IU/mL, Sigma Biosciences, St. Louis, MO, USA) or IL-15 (10 IU/mL, Sigma Biosciences, St. Louis, MO, USA) and TKD peptide (2 *μ*g/mL, Multimmune GmbH, Munich, Germany) from 1 to 6 days.

### 2.1. Analysis of Cell Surface Expression of NK Activatory and Inhibitory Receptors

Flow cytometry was performed on days +1, +4, +5, and +6 after stimulation with interleukin and TKD peptide (day 0) using a standard direct immunofluorescence technique and mouse anti-human monoclonal antibodies conjugated with fluorescein isothiocyanate (FITC), phycoerythrin (PE), and allophycocyanin (APC) on a FACSCalibur (Becton Dickinson, San Jose, USA). The experiment was settled in such a way that unstimulated cells, cells stimulated with interleukin itself (IL-2 or IL-15), and cells stimulated with the combination of interleukin and TKD peptide (IL-2 + TKD or IL-15 + TKD) were analysed at the same time on each day. 

After washing in PBS containing 10% FCS (Sigma Biosciences, St. Louis, MO, USA), single-cell suspension of 0.5 × 10^6^ cells per tube was stained with FITC or APC-conjugated monoclonal antibodies against CD3 (BD Biosciences, Franklin Lakes, NJ, USA; Exbio Prague Ltd., Vestec, Czech Republic), FITC or PE-conjugated monoclonal antibodies against CD56 (BD Biosciences, Exbio), and FITC, PE- or APC-conjugated monoclonal antibodies against appropriate NK cell activatory or inhibitory receptor for 30 min on ice.

Mouse IgG1-FITC, IgG1-PE, and IgG1-APC were used as isotype-matched controls. All those antibodies were purchased from BD Biosciences (Franklin Lakes, NJ, USA), Exbio Prague, Ltd. (Vestec, Czech Republic), and RD Systems, Inc. (Minneapolis, MN, USA). 

The percentage of positive stained cells was determined as the number of positively stained cells minus the number of cells stained with an isotype-matched negative control antibody. The median fluorescence intensity (MFI) was determined to demonstrate changes in cell surface expression of NK activatory and inhibitory receptors on CD3^−^CD56^+^-positive NK cell population. Only 7-amino-actinomycin D (7-AAD, Becton Dickinson, San Jose, USA) negative, viable cells with intact cell membranes were gated and analysed. 

## 3. Results

### 3.1. Analysis of Cell Surface Expression of NK Cell Receptors in Relation to IL-2 and TKD Peptide Treatment

**Figure 1:**
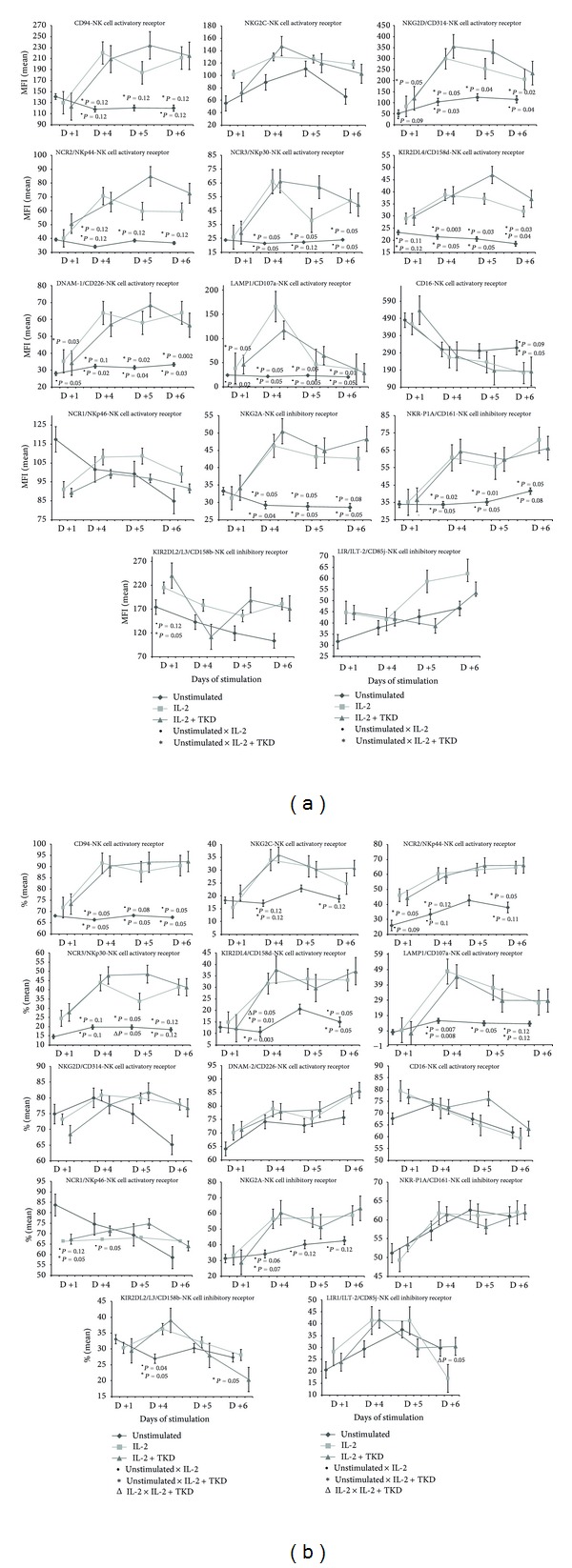
(a) The effect of IL-2 and IL-2/TKD peptide on NK cell surface expression in peripheral blood mononuclear cells of healthy individuals—the amount of receptor expressed by CD3^−^CD56^+^cellular population (the median fluorescence intensity). (b) The effect of IL-2 and IL-2/TKD peptide on NK cell surface expression in peripheral blood mononuclear cells of healthy individuals—the proportion of cells expressing an appropriate receptor.

Compared to unstimulated cells, low-dose IL-2 alone and the combination of IL-2/TKD peptide induced a significant or nearly significant upregulation of CD94, most NK cell activatory receptors such as NKG2D, NKp44, NKp30, KIR2DL4, DNAM-1, LAMP1, and NK cell inhibitory receptors like NKG2A and NKR-P1A (Figure 1(a)). Concurrently, an increase of the proportion of cells expressing an appropriate antigen was observed in case of CD94, NKp44, NKp30, KIR2DL4, LAMP1, and NKG2A (Figure 1(b)). The changes in cell numbers positive for appropriate NK cell receptors or the changes in their cell surface expression were detected from day +1 (NKG2D, NKp44, KIR2DL4, DNAM-1, and LAMP1) or day +4 (CD94, NKp30, NKG2C, NKG2A, and NKR-P1A) till day +6 after the treatment with IL-2 with or without TKD peptide. Although the increase of the positive cell numbers and the median fluorescence intensity for the tested marker were apparent after the treatment with the combination of low-dose IL-2 and TKD peptide when compared to IL-2 alone, it reached a statistical significance just in case of two NK cell activatory receptors (NKp30 and KIR2DL4). 

The diverse effect of IL-2 and IL-2/TKD mixture on cell surface expression was observed in case of NK cell activatory receptors CD16 and NKp46 and NK cell inhibitory receptors KIR2DL2/L3 and LIR1/ILT-2 (Figures 1(a) and 1(b)). Although the proportion of cells expressing CD16 receptor did not significantly change compared to parallel examined unstimulated cells, the continuous decline of the cell surface density was observed with ongoing days of stimulation with IL-2 alone or IL-2/TKD mixture. Opposite situation was observed in case of the receptor NKp46. While the cell surface density of the marker was invariable with regard to various cell culture conditions, a significant decline of cells bearing the surface receptor was detected on day +1 and day +4 after the treatment with IL-2 alone or in combination with TKD peptide compared to unstimulated cells.

The cell number bearing the receptor KIR2DL2/L3 was on day +4 of the treatment with IL-2 or IL-2/TKD significantly increased, but on day +6 significantly decreased. On the other hand, the KIR2DL2/L3 cell surface density was upregulated on day +1 after the treatment with IL-2 w or w/o TKD peptide, but later on days + 4, +5, and +6 it remained unchanged compared to untreated cells.

The cell surface expression and the number of cells expressing LIR1/ILT-2 marker did not differ before and after the stimulation with IL-2 alone or IL-2/TKD mixture. However, a significant increase of cell number positive for LIR1/ILT-2 marker was observed on day +6 of culture with IL-2/TKD peptide mixture compared to IL-2 alone (Figure 1).

### 3.2. Analysis of Cell Surface Expression of NK Cell Receptors in Relation to IL-15 and TKD Peptide Treatment

**Figure 2:**
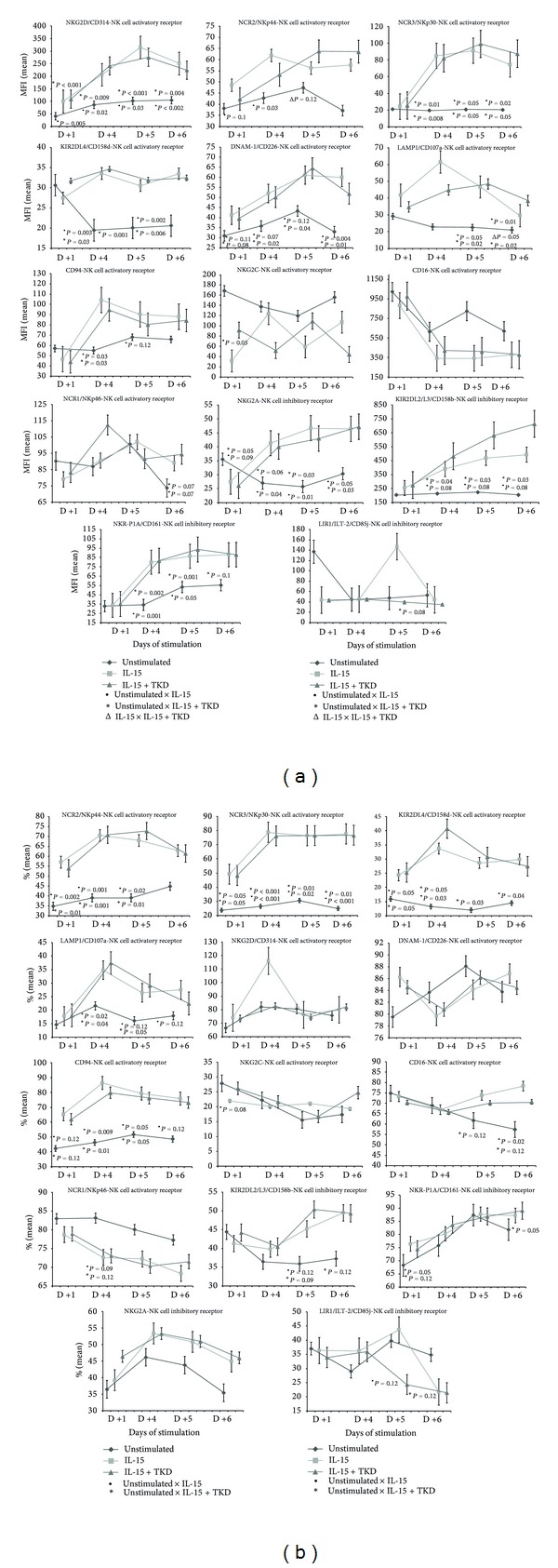
(a) The effect of IL-15 and IL-15/TKD peptide on NK cell surface expression in peripheral blood mononuclear cells of healthy individuals—the amount of receptor expressed by CD3^−^CD56^+^cellular population (the median fluorescence intensity). (b) The effect of IL-15 and IL-15/TKD peptide on NK cell surface expression in peripheral blood mononuclear cells of healthy individuals—the proportion of cells expressing an appropriate receptor.

Stimulation of peripheral blood mononuclear cells with IL-15 alone or IL-15/TKD combination led to upregulation of CD94, NK cell activatory receptors such as NKG2D, NKp44, NKp30, KIR2DL4, DNAM-1, LAMP1, and NK cell inhibitory receptors like NKG2A, KIR2DL2/L3, and NKR-P1A (Figure 2(a)). Upregulated expression of CD94, NKp44, NKp30, KIR2DL4, LAMP1, KIR2DL2/L3, and NKR-P1A receptors was accompanied by increased number of cells positive for the tested marker (Figure 2(b)). Except for LAMP1, NKG2A, and KIR2DL2/L3 receptors, the effect of IL-15 alone or with TKD peptide on the cell surface expression was apparent from day +1 of the culture. The difference in the median fluorescence intensity between IL-15 and IL-15/TKD stimulated cells was observed in LAMP1 and NKp44 receptors.

The diverse effect of IL-15 and IL-15/TKD mixture on cell surface expression was observed in case of NK cell activatory receptors CD16, NKG2C, and NKp46 and NK cell inhibitory receptor LIR1/ILT-2 (Figures 2(a) and 2(b)).

IL-15 alone or IL-15/TKD caused the decline in the cell surface density of CD16 receptor, but the difference between stimulated and unstimulated cells did not reach a statistical significance. On the other hand, the treatment affected positively the number of cells expressing a given marker on days +5 and +6.

While the MFI values of NKp46 receptor were substantially variable on various days of culture, they trended to a significant rise on day +6 of culture in stimulated cells compared to unstimulated cells. Furthermore, the number of cells positive for NKp46 trended to reach a significant decline after the treatment with IL-15 with or without TKD peptide.

Although the proportion of cells expressing NKG2C receptor did not differ between IL-15 and IL-15/TKD stimulated and unstimulated cells, the decline of the cell surface density was observed after the treatment with IL-15 alone or IL-15/TKD mixture, which reached on day +1 a statistical significance.

A trend to the decline in number of positive cells for LIR1/ILT-2 receptor followed by the downregulation of cell surface expression was observed on days +5 and +6 of culture with IL-15/TKD peptide mixture compared to unstimulated cells.

## 4. Discussion

NK cells represent a potential tool for adoptive immunotherapy against tumors [8]. Human NK cells express numerous activatory, coactivatory, and inhibitory receptors which recognize specific ligands expressed by target cells [9]. NK cell unresponsiveness is under the dominant control of inhibitory receptors that bind with ubiquitously expressed MHC class I molecules. On the other hand, NK cell activation and cytotoxicity are mediated by a deviation in the balance between inhibitory and activatory receptors in favour of activatory receptor signalling [10]. Previous studies of Multhoff group [2] demonstrated that membrane-bound Hsp70 acted as a tumor-specific marker enhancing NK cell activity. They subsequently showed that Hsp70-derived 14-mer peptide (TKD) in combination with IL-2 enhances the cytolytic activity of resting NK cells against tumor cells presenting Hsp70 on their cell membrane [3]. Later, in phase I clinical trial they have shown in patients with metastatic colon carcinoma and nonsmall cell lung cancer that the application of ex vivo TKD/IL-2 activated autologous leukapheresis product is safe and maintained the cytolytic activity against autologous tumors [6, 7]. 

In the present study, we analysed cell surface expression of NK cell activatory receptors (CD16, NKG2D, NKG2C, NKp46, NKp44, NKp30, KIR2DL4, DNAM-1, and LAMP1) and NK cell inhibitory receptors (NKG2A, KIR2DL2/L3, LIR1/ILT-2, and NKR-P1A) in relation to peripheral blood mononuclear cell treatment with IL-2 and IL-15 in the presence or absence of hsp70-derived 14-mer peptide (TKD) using flow cytometry. Results were expressed as the percentage of receptor expressing cells as well as the amount of receptor expressed by CD3^−^CD56^+^NK cellular population (MFI). This is the first study demonstrating the effect of IL-2/TKD on cell surface expression of NK cell activatory receptors (KIR2DL4, DNAM-1, and LAMP1) and NK cell inhibitory receptors (LIR1/ILT-2 and NKR-P1A). The cell surface expression of NK cell activatory receptors (KIR2DL4, DNAM-1, and LAMP1) and NK cell inhibitory receptors (KIR2DL2/L3, LIR1/ILT-2, and NKR-P1A) in relation to IL-15/TKD has not yet been also investigated. 

As expected, CD94 receptor and most NK cell activatory receptors (6 out of 9 examined markers: NKG2D, NKp44, NKp30, KIR2DL4, DNAM-1, and LAMP1) were upregulated after the stimulation with IL-2 alone or in combination with TKD peptide. Likewise an increase in a number of NK cells with the appropriate receptor on their cell surface was demonstrated in 6 out of 10 examined markers (CD94, NKp44, NKp30, NKG2C, KIR2DL4, and LAMP1). Surprisingly, IL-2 alone or in mixture with TKD peptide induced also a significant upregulation of 2 out of 4 examined NK cell inhibitory receptors (NKG2A and NKR-P1A). On the other hand, CD16 receptor was downregulated after IL-2 or IL-2/TKD actions. Our data are in agreement with the data of Stangl et al. [11] who examined as well the cell surface expression of CD94, NKG2D, NKp44, NKp30, NKp46, NKG2C, NKG2A, and CD16 markers in peripheral blood lymphocytes cultured with IL-2 alone and IL-2/TKD mixture. They reported a significant upregulation in the cell surface density of CD94, NKG2C, NKG2D, NKp30, NKp44, and NKG2A and downregulation of CD16 receptor caused by IL-2 itself [11]. However, the results derived from our study differ from those reported by Stangl et al. [11] in case of NKp46 receptor. While Stangl et al. [11] observed an upregulation of NKp46 receptor after IL-2/TKD incubation lasting for 5 days, we demonstrated no effect of IL-2 alone or IL-2/TKD mixture on day +5 of the culture. Moreover, our study demonstrated a decrease of cell number positive for NKp46 marker on day +1 and day +4 of stimulation with IL-2 w or w/o TKD peptide.

This study confirmed also the data of Chrul et al. [12] referring to the fact that the proportion of cells expressing NK cell inhibitory receptor KIR2DL2/DL3 is also significantly increased after 3 days of stimulation with IL-2 alone. We observed the rise of cell numbers positive for KIR2DL2/DL3 marker not only after IL-2 alone treatment, but also after IL-2/TKD treatment on day +4 of the culture. But it seems that the increase of cell number bearing KIR2DL2/DL3 marker reached the peak values just between the days +3 and +4 of stimulation and was followed by a significant decline on day +6 of the culture. In case of NK cell inhibitory receptor LIR1/ILT-2 no effect of IL-2 alone or in combination with TKD peptide was observed on cell surface expression before day +5 of stimulation.

Likewise in case of IL-2 and IL-2/TKD stimulation of peripheral blood mononuclear cells with IL-15 and IL-15/TKD showed upregulation of the same NK cell activatory and inhibitory receptors with the exception of NK cell inhibitory receptor KIR2DL2/L3 which was upregulated only by IL-15 and IL-15/TKD. The proportion of cells was the same that as in case of IL-2 and IL-2/TKD treatment also increased in CD94, NKp44, NKp30, KIR2DL4, and LAMP1 receptors. Moreover, IL-15 and IL-15/TKD combination caused a cell number rise positive for KIR2DL2/L3 and NKR-P1A receptors. On the other hand, it had no effect on cell number positive for NKG2C and NKG2A receptors that were positively influenced only by IL-2 and IL-2/TKD combination. These data confirmed partially the data of Stangl et al. [11] who observed as well a significant increase in the cell surface density of CD94, NKG2D, NKp30, NKp44, and NKG2A after the treatment with IL-15 and TKD peptide. However, our data differ from that of Stangl et al. study [11] in case of NKG2C and NKp46 receptors whose upregulation by IL-15 and IL-15/TKD was not demonstrated by our group.

Analogous to Stangl et al. [11] CD16 cell surface expression was downregulated by IL-2 or IL-2/TKD action, but contrary to Stangl et al. [11] CD16 cell surface downregulation did not reach after the treatment with IL-15 or IL-15/TKD, a statistical significance in our study. Moreover, IL-15 or IL-15/TKD treatment led to the increase of cell number positive for CD16 marker, which IL-2 alone or IL-12/TKD combination did not elicit. Contrary to IL-2/TKD treatment, IL-15/TKD combination showed strong negative effect on NK cell inhibitory receptor LIR1/ILT-2, whose expression was downregulated.

The expression of NK cell receptors is largely transcriptionally and posttranscriptionally regulated [13]. IL-2, IL-12, and IL-15 induce similar, yet distinct functional effects on NK cells [14]. For instance IL-2 utilizes members of the Janus protein tyrosine kinase (Jak) family and activates molecules of the STAT family, which bind selective DNA sequences [15–17]. Moreover, IL-2 may induce expression of *c-fos*, *junB*, *egr-1*, and *bcl-2* genes, which modulate expression of NK cell recognition structures and/or other effector cell molecules involved in triggering cytotoxicity [14, 18]. 

Fc*γ*RIII (CD16) is a receptor for complexed IgG. Two very homologous genes, located on chromosome 1 in humans, coding for this receptor, are expressed in a cell type-specific way. The *FCGR3A* gene, which encodes Fc*γ*RIIIa, is expressed as a transmembrane protein by NK cells and macrophages, whereas the polymorphic *FCGRIIIB* gene is constitutively expressed only by neutrophils and can be induced on eosinophils by IFN*γ* [19, 20]. Fc*γ*RIIIa/CD16a is involved in antibody-dependent cell-mediated cytotoxicity (ADCC). Reduced antibody-dependent cellular cytotoxicity in response to TGF-*β*1 was observed in human NK cells [21]. Whereas most of the reports focus on the regulation of CD16 expression in neutrophils and myeloid cells; no data are available for NK cell population. However, previous data suggest that the phosphatidylcholine-specific phospholipase C (PC-PLC) enzyme could play an important role in regulating the CD16 membrane expression, the CD16-mediated cytolytic mechanism, and the CD16-triggered signal transduction in NK cells. PC-PLC and CD16 distribution in NK cell plasma membrane demonstrates that the proteins are physically associated and partially accumulated in lipid rafts [22].

The KIR and ILT/LIR protein families, whose genes are located within the human LRC, are structurally and functionally comparable. The molecular mechanisms that regulate the clonally diverse expression of KIR and LIR genes on NK cells are not known [23]. It is possible that regulation of LIR expression in NK cells shares some features with highly related KIR receptors [23]. It was demonstrated that PU.1 (Spi-1) plays a cruial role in ILT transcription [24]. To a lesser extent, Sp1 family transcription factors also affected transactivation of ILT2/4 [25]. The protein kinase C (PKC) family, which is activated by phosphatidylserine and diacylglycerol via Ca^2+^-dependent manner or by PMA (phorbol ester) [26–28], was described to be involved in upregulation of inhibitory killer Ig-like receptor surface expression [29, 30]. Additionally, it has been suggested that epigenetic mechanisms might be responsible for establishing and maintaining differential KIR and LIR expression patterns [31, 32]. While DNA methylation plays a role in regulation of KIR expression and may result in monoallelic expression [33, 34], ILT expression is tightly regulated by histone acetylation [32]. Moreover, allelic polymorphism of some KIR and LIR, involving KIR2DL4 and ILT2 genes, was found to correlate with transcriptional activity and surface protein expression [23, 32, 35, 36]. 

Similarly, the mechanisms regulating NCR expression on NK cells are not yet well understood [37]. However, the study of [38] has demonstrated the possibility that NCR expression could also be regulated by PKC in NK cells. Prolactin induces upregulation of NCR expression augmenting NK cytotoxicity against tumour cells, and vice versa corticosteroids or TGF-*β*1 reduces NK cytotoxicity [39, 40]. A recent study of [37] revealed that the conformation of PKC-binding sites might result in posttranscriptional regulation of NKp46 expression. In experiments with mutant NKp46-expressing stable cell lines they demonstrated that the amino acid sequence motif (Ser288) in the cytoplasmic tail of NKp46 might be critical for PKC-mediated regulation of NKp46 cell surface expression. Likewise in our experiments, they also demonstrated that IL-2 did not induce any change of NKp46 expression on NK cells in contrast to NKp44 which is upregulated during in vitro culture with IL-2 [41, 42]. 

The genes encoding NKG2D, CD94/NKG2, and NKR-P1 receptors are localized in the NK complex on chromosome 12 [43–45]. NKG2D is expressed very early in the development of NK cells; its expression rapidly increases and remains high through all later stages of maturation [46–48]. IL-15 is an essential cytokine for the development and survival of NK cells [49, 50]. The intracellular signalling components of IL15R and NKG2D have been shown to be coupled [51]. Jak3, as a part of canonical IL15-R signalling pathway, is responsible for the phosphorylation of DAP10 important for the activation of STAT5. Activated DAP10 recruits also PI3K and Grb2, which control proliferation, survival, and cytotoxicity of NK cells [52, 53]. NKG2D receptor was significantly upregulated after the treatment with IL-2 and IL-15 and downregulated after culture with TGF-*β* [54, 55]. Likewise as in case of KIR and LIR genes, epigenetic mechanisms, such as DNA methylation and histone acetylation, participate in NKG2D gene regulation in T lymphocytes and NK cells [56]. 

The human *cd94* gene has two promoters, encoding the same CD94 protein, with differential sensitivity to IL-2 and IL-15 [57]. Usually the proximal promoter is active in NK and CD8+ T cells, but after exposure of cells to IL-2 or IL-15 the distal promoter quickly becomes active [58]. In case of the *nkg2a* gene the transcription initiates from multiple starting sites [59]. IL-2, IL-15, IFN-*α*, and IL-21 have been demonstrated to induce the expression of CD94/NKG2A in NK and T cells [13, 60–63]. Little is known about the biologic role and the expression of the CD94/NKG2C dimer during NK and T-cell differentiation [64]. However, both *nkg2a* and *nkg2c* genes may be cotranscribed at the clonal level in some NK and T cells, and both proteins may be detected together at the surface of decidual and peripheral blood CD56^bright^ NK cells [65–67]. The functional implications resulting from coexpression at the single-cell level of activating and inhibitory receptors specific for the same ligand are unknown [64]. In our study, CD94 and NKG2A receptors were upregulated after the stimulation with IL-2 or IL-15 alone or in combination with TKD. Simultaneously, cell number rise positive for CD94 was observed. Only IL-2 and IL-2/TKD increased the number of cells bearing NKG2C and NKG2A receptors. 

Our data are not in compliance with that of the study of [14], who showed that the expression of NKR-P1A/CD161 was specifically upregulated by IL-12, while other cytokines (IL-2 and IL-15) did not mediate these effects. They try to explain this observation by differential gene transcription regulation. A novel gene, 197/15a, was found to be downregulated by IL-2 and IL-15 and upregulated by IL-12 in NK and T cells. However, the mechanisms of transcription regulation of NKR-P1A/CD161 are still poorly understood, and therefore it is too early to make final conclusion [68]. 

DNAM-1/CD226 gene has been mapped to human chromosome 18 [69]. The mechanism of DNAM-1/CD226 gene regulation is still unknown. But, it was shown that DNAM-1/CD226 gene and surface expression might be stimulated by treatment of the cells with phorbol ester (TPA) through the activation of gene promoters P1 and P2 by activating protein-1 (AP-1) [70]. IL-2 and TNF-*α* augment DNAM-1/CD226 expression and cytotoxic function of effector cells as well, whereas TGF-*β* could inhibit both of these events. Moreover, evidence was provided that the IRF-1, an IFN-*γ*-induced transcription factor pivotal in the regulation of infection and inflammation, upregulated TRAIL and DNAM-1/CD226 on NK cells. Mechanistic investigations revealed that IRF-1-induced NK cell cytotoxicity was independent of perforin and granzyme B but dependent on the NK-cell-activating receptor DNAM-1 [71]. Recent studies also demonstrated the importance of the 3′-UTR SNP rs727088 in the regulation of DNAM-1/CD226 transcription in T and NKT cells and its association with autoimmunity [72]. 

Finally, LAMP1/CD107a provides information of the activation level of the effector population. It was shown that it is upregulated on NK cells following stimulation and its expression correlates with cytokine secretion and NK-cell-mediated lysis of target cells [73]. LAMP1/CD107a expression on NK and T cells was shown to be significantly increased after IL-2 stimulation. That is why LAMP1/CD107a represents a sensitive candidate marker for the evaluation of cytotoxic activity [74].

## 5. Conclusion

In conclusion, although NK cell cytotoxicity finally depends on the type and the expression of ligands on the target cells interacting with NK cell activatory and inhibitory receptors [9, 10], the study showed clearly how NK cells were influenced under in vitro conditions in the presence of other immunocompetent cells by low-dose interleukins themselves or in combination with TKD peptide.
